# Supplementary material for: Wheat microbiome bacteria can reduce virulence of a plant pathogenic fungus by altering histone acetylation
Source: Nat Commun. 2018 Aug 24;9:3429. doi: 10.1038/s41467-018-05683-7 (PMC6109063; doi:10.1038/s41467-018-05683-7)
Supplement: Supplementary file 1 — Supplementary Information [file 41467_2018_5683_MOESM1_ESM.pdf]

Wheat microbiome bacteria can reduce virulence of a plant pathogenic fungus by altering histone acetylation

Yun Chen et al.

Supplementary information

Supplementary Figures

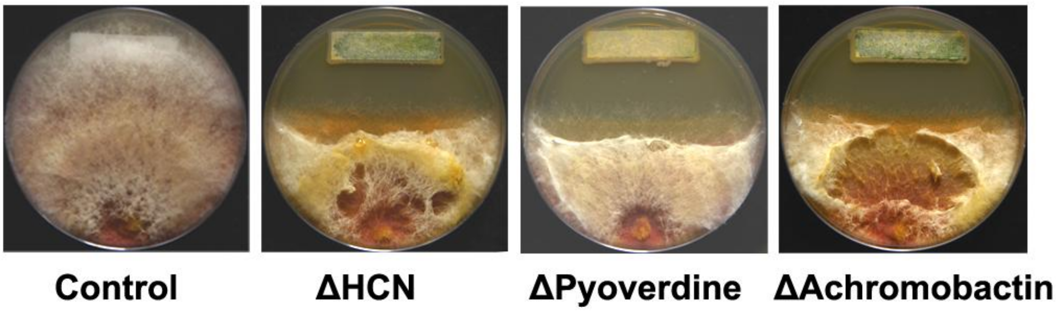

Supplementary Figure 1. Antagonistic activities of hydrogen cyanide (HCN), pyoverdine and achromobactin biosynthetic cluster mutants towards *F. graminearum* in a co-culture assay.

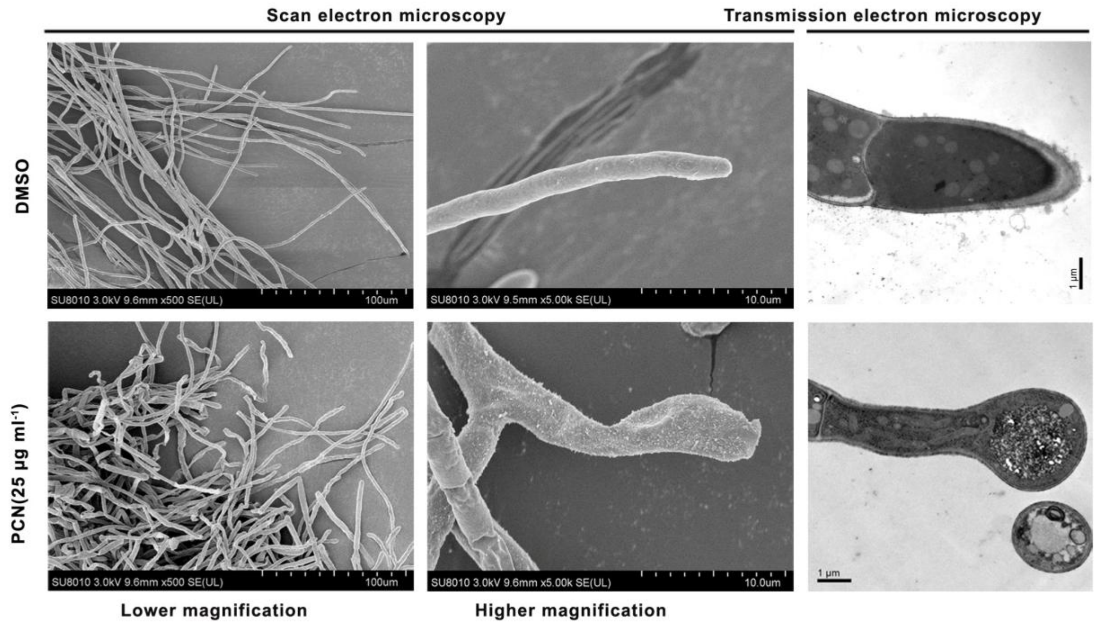

Supplementary Figure 2. Morphological characteristics of PCN-treated *F. g* hyphae. Ultrastructural morphology of *F. g* hyphae treated with PCN. Images were obtained by scanning electron microscopy (SEM) (left-hand and middle panels) and transmission electron microscopy (TEM) (right-hand panel).

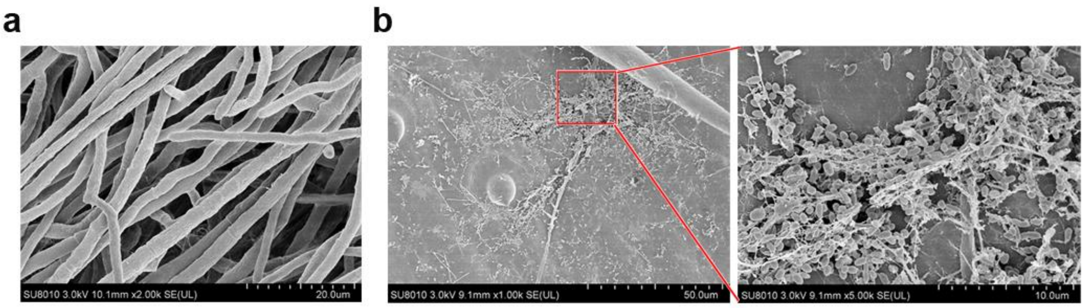

Supplementary Figure 3. Morphology of F. g grown in WA and ZJU60 *in planta*. **a.** Hyphal morphology of *F. graminearum* grown in WA broth without PCN treatment. **b.** Biofilm-like macroconoly features of ZJU60 on the surface of a wheat head. The cell suspension of ZJU60 was sprayed on the wheat heads. After 7 days of incubation, the plant samples were fixed and observed by a scanning electron microscopy.

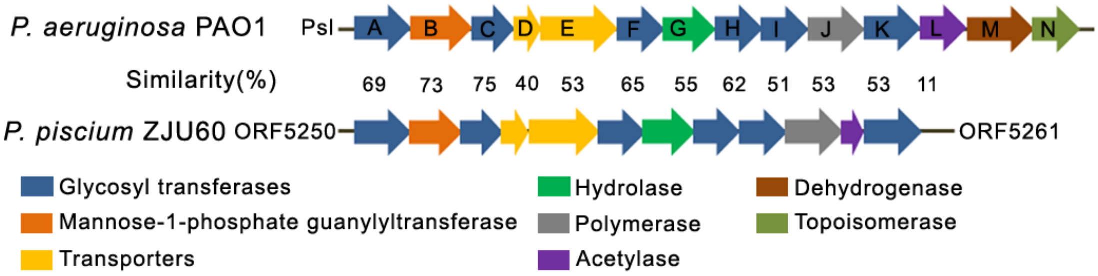

Supplementary Figure 4. Arrangement of the *psl* operon in ZJU60 and the comparable region in *P. aeruginosa* PAO1. PslA-O proteins are shown in the color corresponding to the putative functions assigned to the gene products in the lower panel. Amino acid similarities of counterpart proteins are indicated.

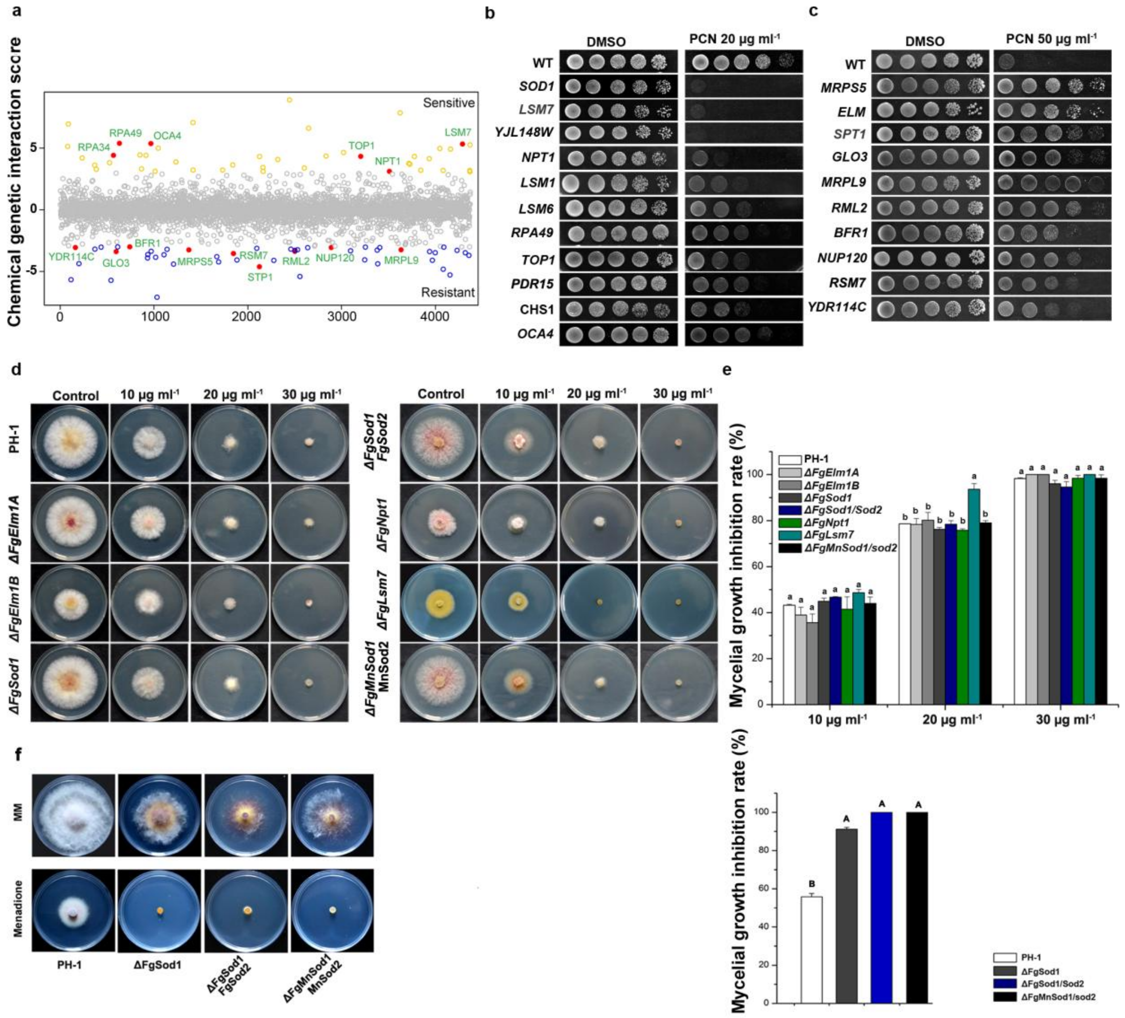

Supplementary Figure 5. Chemical genomic analyses of PCN against *S. cerevisiae*. **a**. The chemical genetic interaction score for each mutant was calculated and plotted. The gene deletion mutants with significant growth changes between PCN treated and control samples are marked in yellow (sensitive to PCN) or blue (resistant to PCN) circles. Red colored circles represent the genes that were experimentally validated in this study, and gene names are shown in green color; **b**. Yeast mutants exhibited significantly increased susceptibility to PCN; **c**. Yeast mutants exhibited significantly increased resistance to PCN; **d**. Sensitivity of deletion mutants of yeast gene homologs in *F. graminearum* to PCN at various concentrations. **e**. The mycelial growth rate was calculated and statistically analyzed for wild-type and mutants. **f**. Sensitivity of deletion mutants of superoxide dismutases in *F. graminearum* towards menadione. The wild-type and mutant strains were grown on the MM and MM amended with menadione at 50 µM, respectively. The mycelial growth rate was calculated and statistically analyzed for the wild-type strain and

mutants. Data presented are the mean ± s.d (n=3).The same letter on the bars for each column indicates no significant difference according to a LSD test at *P* = 0.01.

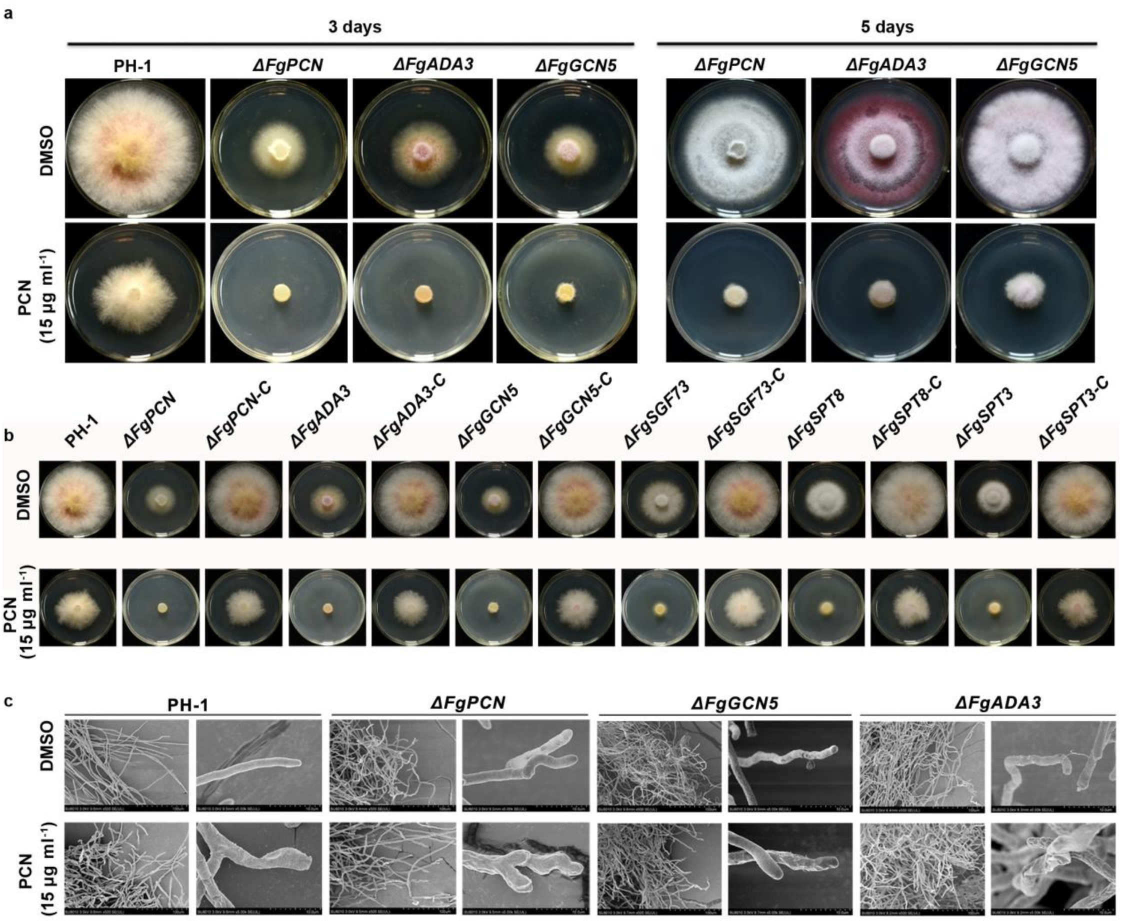

Supplementary Figure 6. Phenotypes of mutants of key genes of the SAGA complex to PCN. a. Sensitivity of the *ΔFgPCN*, *ΔFgADA3*, and *ΔFgGCN5* and wild-type strain towards PCN at 15 µg ml^{-1}. Images were taken after 3 (left panel) or 5 days (right panel) of incubation. b. PCN sensitivity of deletion mutants of **s**ix key components of the SAGA complex and their complemented strains. Images were taken after 3 days of incubation. **c**. Hyphal morphology of *ΔFgPCN*, *ΔFgADA3*, and *ΔFgGCN5* mutants and the wild-type treated with PCN or the solvent control. Mycelia were fixed and observed with scanning electron microscopy (SEM).

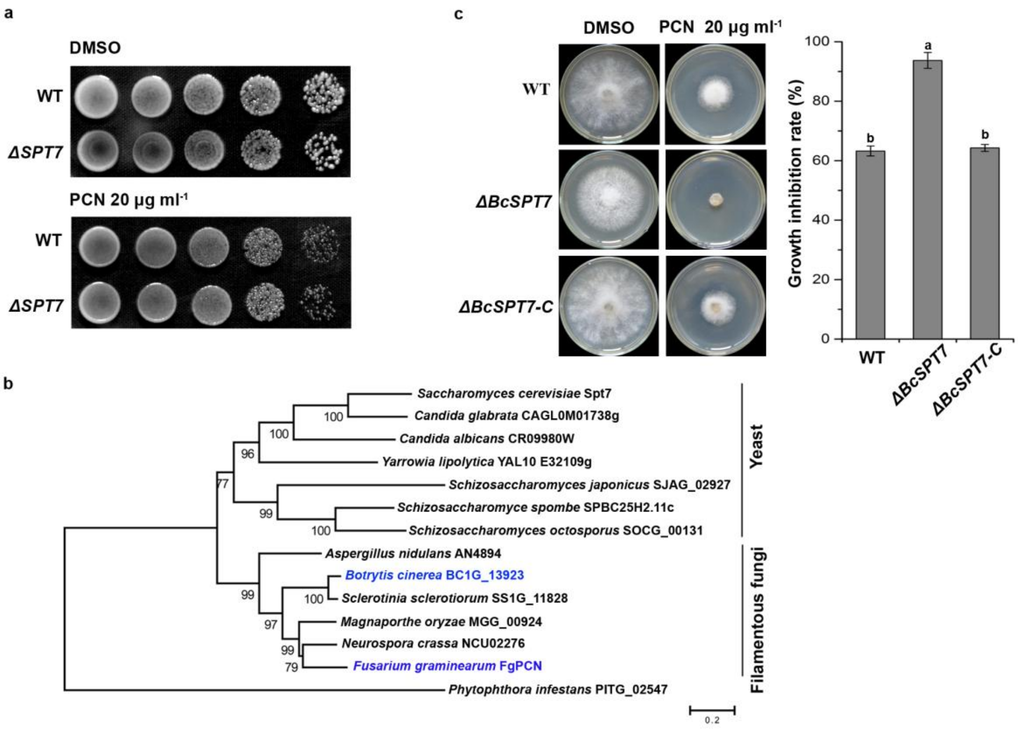

Supplementary Figure 7. Antifungal action of PCN against filamentous fungi may be conserved by targeting the SAGA complex. **a.** Sensitivity of yeast SPT7 (the homolog of FgPCN) mutant to 20 μg ml^{-1} PCN. **b**. Phylogenetic tree of fungal Spt7 homologous proteins. The tested fungi and accession numbers of their SPT7 homologs are indicated. *Phytophthorainfestans* PITG_02547 was used as an out-group control. **c**. Sensitivity of the *Botrytis cinerea BcSPT7* mutant to 20 μg ml^{-1} PCN.The mycelial growth rate was calculated and statistically analyzed. Data presented are the mean ± s.d (n=3).The same letter on the bars for each column indicates no significant difference according to a LSD test at *P*=0.05.

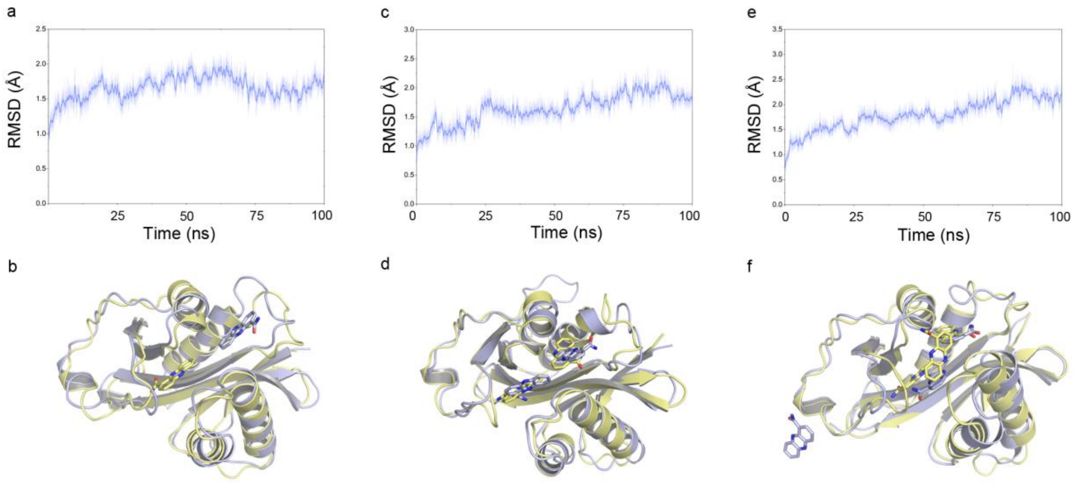

Supplementary Figure 8. Molecular dynamics simulation of docked binding positions. The time evolution of the root mean square deviations (RMSDs) of the predicted binding positions of PCN at FgGcn5 with binding ratios of 1:1(a), 1:2(c), and 1:3(e). Alignment of the initial structure (light green) and last snapshot (light purple) from the molecular dynamics (MD) simulation of PCN at FgGcn5 with binding ratios of 1:1(b), 1:2(d), and 1:3(f).

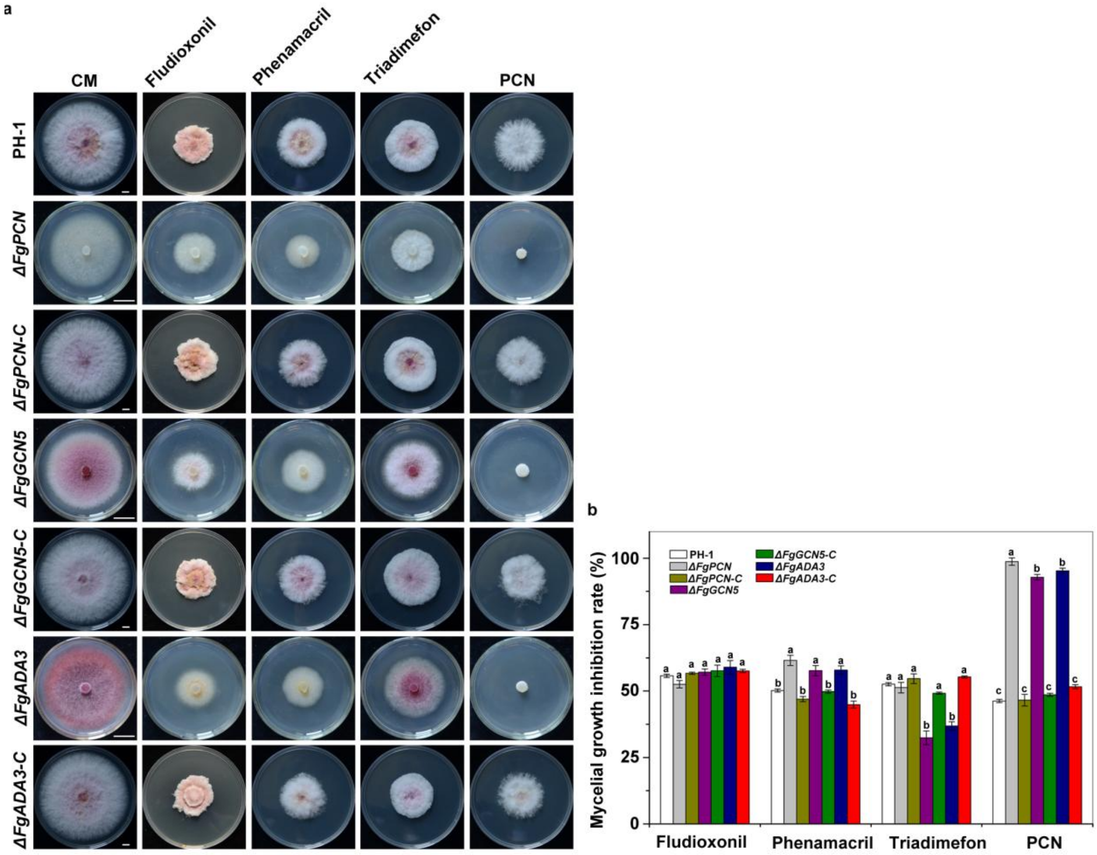

Supplementary Figure 9. Sensitivity of *ΔFgPCN*, *ΔFgADA3* and *ΔFgGCN5* to tested fungicides. **a**. Drug sensitivity images were obtained after 6 days of incubation at 25 °C on PDA plates containing fludioxonil, phenamacril, triadimefon or PCN at a final concentration of 50% for a maximal effect (EC$_{50}$=0.1,0.25,5 and 15 µg ml^{-1}, respectively). Bars indicating plate size are 1 cm. **b**. The mycelial growth inhibition rate was calculated after 6 days of incubation. Data presented are the mean ± s.d (n=3).The same letter on the bars for each column indicates no significant difference according to a LSD test at *P*=0.05.

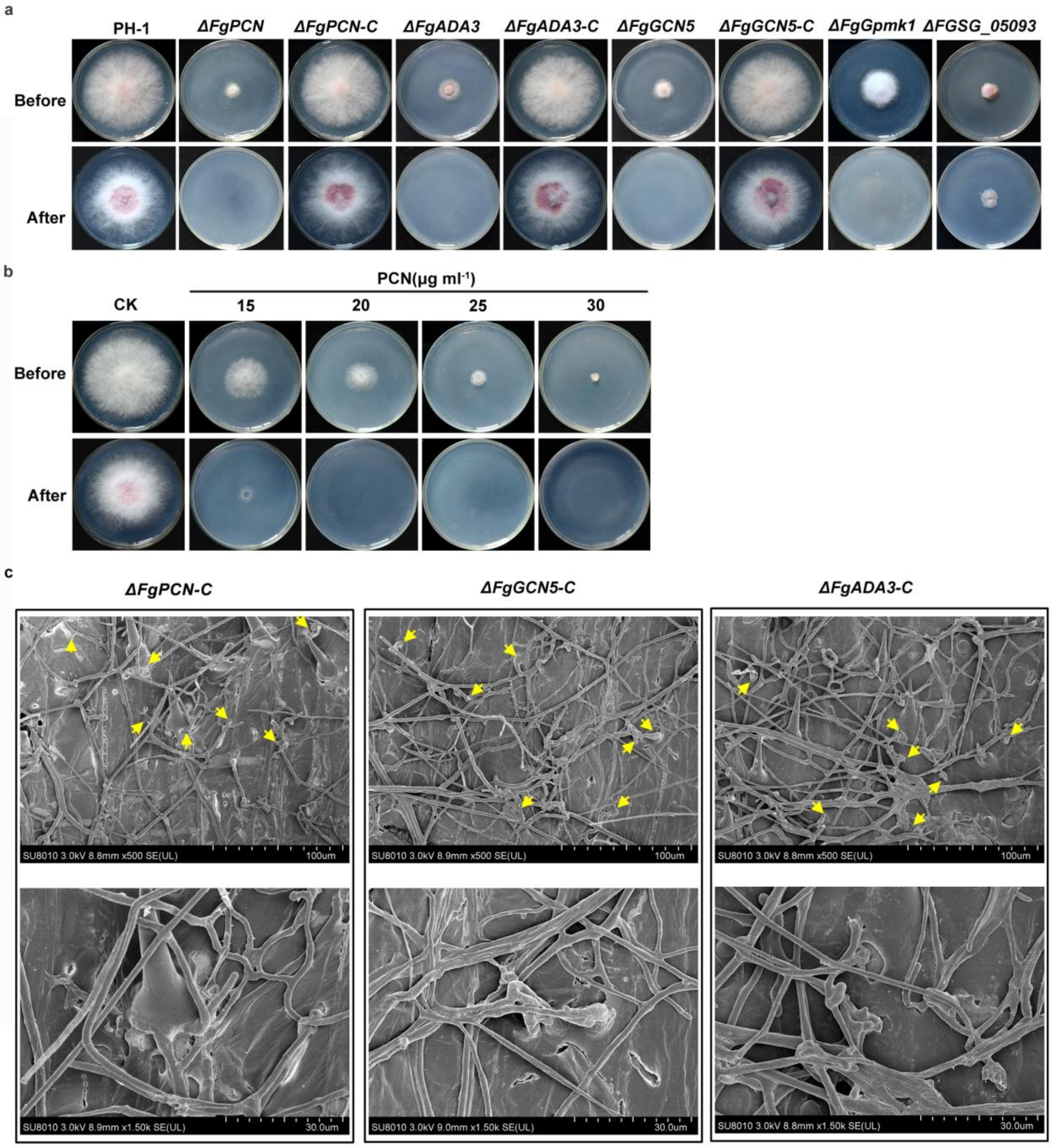

Supplementary Figure 10. Penetration of strains into cellophane sheets and wheat glumes. **a**. Fungal colonies were grown for 2 days at 25 °C on top of cellophane membranes placed on minimal medium (Before). The cellophane membranes with the fungal colonies were removed, and the plates were incubated for an additional 3 days to examine the presence of mycelial growth on the plate, indicating penetration of the cellophane (After). The *ΔFgGpmk1* mutant was used as a non-penetrated control. The *ΔFGSG_05093* mutant was used as a control to exclude the growth defect. b. The penetration ability of the wild-type in the present of PCN. The final concentration of PCN was indicated in figure. **c**. Infection structures of complemented strains on dissected wheat glumes. Wheat head spikelets were inoculated with mycelium of each strain. Samples were taken 72 h after inoculation, and examined with the scanning

electron microscopy. The red arrows indicate the typical infection structures of the fungus.

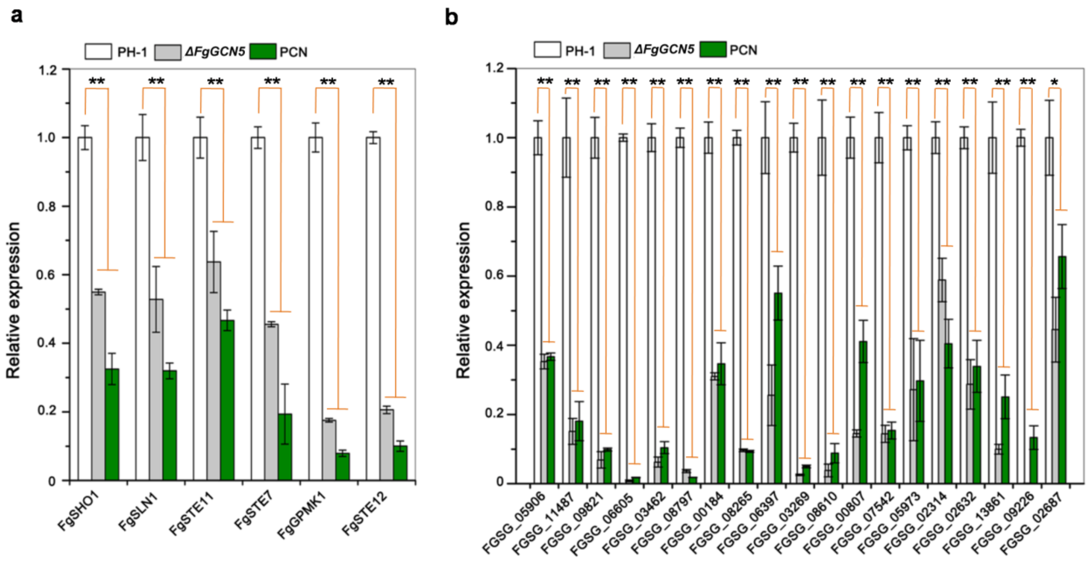

Supplementary Figure 11. Relative expression of penetration genes in *ΔFgGCN5* and in the present of PCN. a. Relative expression levels of penetration-related genes in the FgGpmk1 mitogen-activated protein kinase cascade in the wild-type PH-1, *ΔFgGCN5* and PCN treatment (25 μg ml^{-1}). **b**. Relative expression levels of the representative genes encoding plant cell wall degrading enzymes in the wild-type PH-1, *ΔFgGCN5* and PCN treatment (25 μg ml^{-1}). Mycelia were collected after 48 h grown in CM medium and the gene expression levels were analyzed by qRT-PCR. Relative expression levels were normalized with actin as an internal standard and presented as means ± s.d (n = 3). Asterisks represent significant differences between the wild type and *ΔFgGCN5* or PCN treatment according to a LSD test at *P*=0.01.

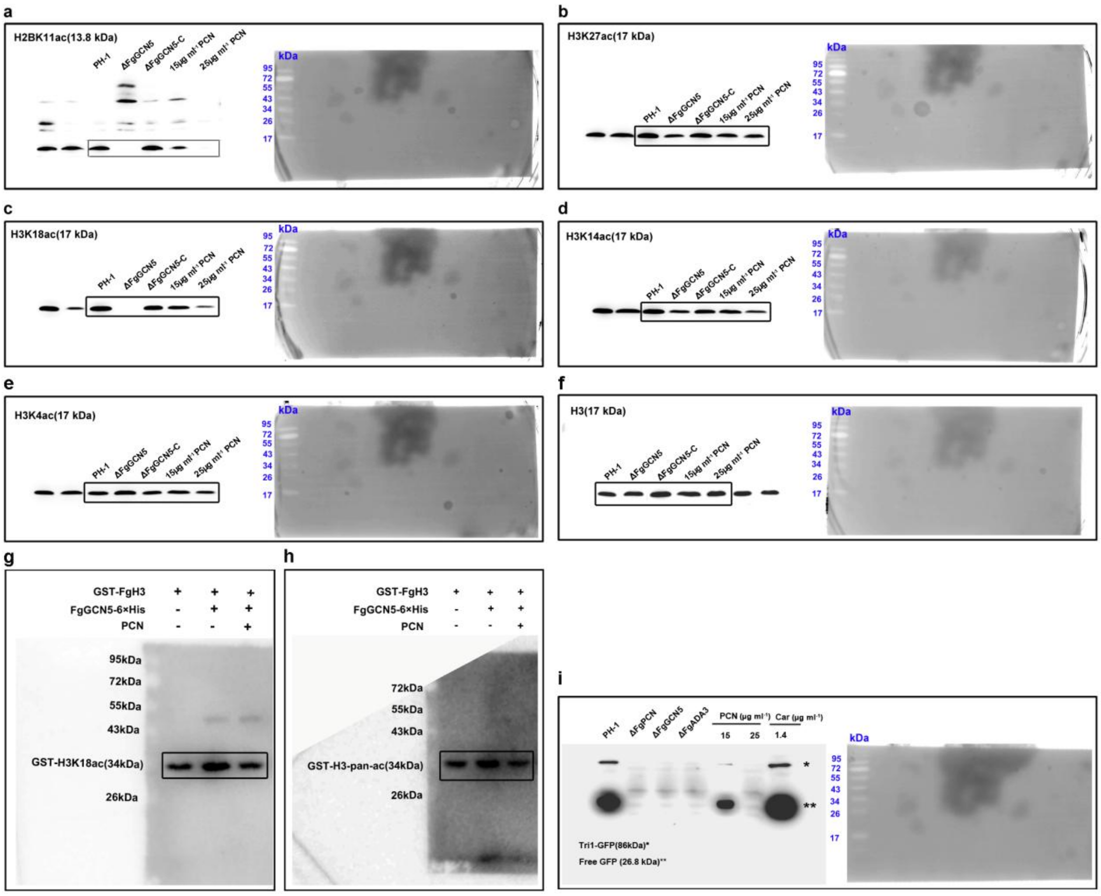

Supplementary Figure 12. Uncropped scans of the most important western blots. **a-e**. Effects of FgGcn5 deletion and PCN treatment on histone acetylation at H2BK11, H3K27, H3K18, H3K14 and H3K4. **f**. Protein loading control was blotted with an anti-H3 C-terminus antibody. **g-h**. Inhibition of activity against the acetyltransferase activity of FgGcn5 on H3K18 and H3 *in vitro*. The H3K18ac and H3-pan-ac antibody was used for immunoblotting, respectively. **i**. Expression of Tri1-GFP in tested mutants and the wild type treated with PCN or Carbendazim was verified by immunoblot assays using the anti-GFP antibody.

Supplementary Table 1. Relative abundances of the dominant genera in the wheat head microbiome

Genus	Relative abundances (%)		Fold change
	Healthy	Infected	
Pseudomonas	0.31	2.93	9.58
Comamonas	0.03	0.14	5.07
Paenibacillus	0.06	0.23	4.05
Erwinia	1.67	4.53	2.72
Hymenobacter	2.64	6.98	2.65
Achromobacter	0.14	0.34	2.47
Methylobacterium	1.53	3.29	2.16
Sphingomonas	10.76	17.35	1.61
Luteibacter	0.00	1.55	
Chryseobacterium	0.00	1.01	
Agrobacterium	0.00	1.69	
Frigoribacterium	0.00	1.89	
Pedobacter	0.00	0.79	
Rathayibacter	0.00	0.08	
Mycetocola	0.00	0.96	
Acinetobacter	0.00	0.03	
Burkholderia	0.00	0.14	
Fusobacterium	0.00	0.06	
Variovorax	0.00	0.17	
Kineococcus	0.00	1.01	
Bacillus	0.00	0.93	
Rhodococcus	0.00	0.03	
Aeromicrobium	0.00	0.06	
Prevotella	0.00	0.06	
Saccharibacillus	0.00	0.17	
Pantoea	0.75	0.79	1.05
Lactobacillus	0.03	0.03	1.01
Janthinobacterium	0.03	0.03	1.01
Curvibacter	0.08	0.08	1.01
Deinococcus	0.28	0.00	0
Ralstonia	0.31	0.25	0.83
Ochrobactrum	0.22	0.14	0.63
Polaromonas	0.72	0.37	0.51
Nevskia	0.23	0.08	0.34
Microbacterium	0.25	0.08	0.34

Supplementary Table 2. Summary of screening results for antagonistic isolates obtained from healthy and infected wheat heads

	Total of tested isolates	Antagonistic isolates	Radius of inhibition zone toward Fg (mm)		
			<5	5–10	>10
Number	12,854	492	254	148	90
Percentage		3.82%	1.98%	1.15%	0.70%

Supplementary Table 3. Identification of ZJU60 by gas chromatography of cellular fatty acid

RT	Response	Ar/Ht	RFact	ECL	Peak Name	Percent
1.573	4.686E+8	0.024	----	7.031	SOLVENT PEAK	----
1.952	531	0.023	----	7.814		----
2.188	453	0.021	----	8.301		----
2.499	1149	0.021	----	8.943		----
2.934	723	0.038	----	9.841		----
3.004	1121	0.026	----	9.985		----
3.326	913	0.024	----	10.471		----
3.627	117	0.018	1.132	10.919	Sum In Feature 2	0.04
3.853	465	0.026	----	11.193		----
4.056	13974	0.025	1.093	11.421	10:0 3OH	4.06
4.572	6298	0.034	1.055	12.000	12:0	1.77
4.965	956	0.028	----	12.350		----
5.116	619	0.029	1.029	12.485	unknown 12.484	----
5.159	302	0.025	----	12.524		----
5.930	18324	0.032	0.998	13.176	12:0 2OH	4.85
6.079	1579	0.032	0.993	13.288	12:1 3OH	0.42
6.302	13596	0.034	0.987	13.454	12:0 3OH	3.56
6.777	337	0.032	----	13.809		----
7.034	2460	0.033	0.969	14.000	14:0	0.63
9.000	639	0.034	----	15.274		----
9.351	823	0.046	0.938	15.487	Sum In Feature 2	0.21
9.896	119411	0.040	0.933	15.818	Sum In Feature 3	29.60
10.198	145481	0.042	0.931	16.002	16:0	35.98
11.718	25775	0.042	0.923	16.889	17:0 cyclo	6.32
13.348	48597	0.044	0.915	17.823	Sum In Feature 8	11.81
13.657	3147	0.047	0.914	17.999	18:0	0.76
----	940	---	----	----	Summed Feature 2	0.24
----	-----	---	----	----		----
----	119411	---	----	----	Summed Feature 3	29.60
----	48597	---	----	----	Summed Feature 8	11.81

Matches:

Similarity Index	Entry Name
0.745	*Pseudomonas chlororaphis/aureofaciens/aurantiaca*
0.556	*Pseudomonas putida* biotype B/*vancouverensis*
0.543	*Pseudomonas agarici*
0.459	*Pseudomonas fluorescens* biotype G/*taetrolens*

Supplementary Table 4. General genome features of the 8 biocontrol *Pseudomonas chlororaphis* strains

Strain	ZJU60	PCL1391	PCL1606	HT66	GP72	O6	30-84	PB-St2
Bioproject Accession	PRJNA 436763	PRJNA 285287	PRJNA 263136	PRJNA 207007	PRJNA 77215	PRJNA 67531	PRJNA 260498	PRJNA 225665
Features								
Chromosome size (Mbp)	6.81	6.86	6.66	7.30	6.66	6.98	6.66	6.59
G+C content (%)	62.80	62.80	64.01	62.60	62.89	62.80	62.90	63.05
RNA genes	102	80	157	147	88	142	156	72
rRNA genes	16	17	16	12	7	10	19	7
tRNA genes	67	59	71	57	61	60	69	61
Other RNA genes	19	4	70	78	20	72	68	4
Protein-coding genes	5971	5976	6107	6455	6005	6223	5848	5903
Contig(s)	1	17	2	50	347	30	13	23

Supplementary Table 5. Putative antimicrobial metabolites produced by *Pseudomonas chlororaphis* group biocontrol agents

Strain	HCN	PHZ	PRN	HPR	DAPG	PLT	Orfamide	Pyoverdine	Achromobactin
ZJU60	+	PCN, PCA	-	-	-	-	-	+	+
PCL1391	+	PCN, PCA	-	-	-	-	-	+	+
PCL1606	+	-	+	+	-	-	-	+	+
HT66	+	PCN, PCA	+	-	-	-	+	+	+
GP72	+	PCA, 2-OH-PCA	+	+	-	-	-	+	+
O6	+	PCA, 2-OH-PCA	+	+	-	-	-	+	+
30-84	+	PCA, 2-OH-PCA	+	+	-	-	-	+	+
PB-St2	+	PCA, 2-OH-PCA	-	-	-	-	-	+	+
Pf-5	+	-	+	-	+	+	+	+	+

Note: HCN = hydrogen cyanide, PHZ = phenazine, PRN = pyrrolnitrin, HPR = 2-hexyl, 5-propyl resorcinol, DAPG = 2, 4-diacetylphloroglucinol, PLT =pyoluteorin, PCN= phenazine-1-carboxamide, PCA=phenazine-1-carboxylic acid, 1-HP= 1-hydroxyphenazine and 2-OH-PCA= 2-hydroxyphenazine-1-carboxylic acid. The *Pseudomonas protegens* Pf-5 was used as an out-of-specie control.

Supplementary Table 6. Yeast mutations with altered susceptibility to PCN

Genes	Susceptibility	Gene function
**Increased sensitivity**		
SOD1	+++++	Cytosolic copper-zinc superoxide dismutase
LSM7	+++++	Cytoplasmic Lsm1p complex involved in mRNA decay
YJL148W	+++++	RNA polymerase I subunit A34.5
NPT1	+++++	Nicotinate phosphoribosyltransferase
LSM1	+++	Cytoplasmic Lsm1p complex involved in mRNA decay
LSM6	+++	Cytoplasmic Lsm1p complex involved in mRNA decay
RPA49	+++	RNA polymerase I subunit A49
TOP1	+++	Topoisomerase I
PDR15	+++	Plasma membrane ATP binding cassette transporter
CHS1	+++	Chitin synthase I
OCA4	++	Protein required for replication of Brome mosaic virus
**Increased resistance**		
MRPS5	+++++	Mitochondrial ribosomal protein of the small subunit
ELM1	+++++	Serine/threonine protein kinase
STP1	+++++	Transcription factor
GLO3	+++++	ADP-ribosylation factor GTPase activating protein
MRPL9	+++++	Mitochondrial ribosomal protein of the small subunit
RML2	+++++	Mitochondrial ribosomal protein of the large subunit
BFR1	++++	mRNP complexes associated with polyribosomes
NUP120	++++	Subunit of Nup84p complex of nuclear pore complex
RSM7	++++	Mitochondrial ribosomal protein of the small subunit
YDR114C	++++	Unknown function

Supplementary Table 7. Orthologs of SAGA complex subunits in *F. graminearum*

**SAGA module**	***S. cerevisiae***	***F. graminearum***	**Percentage positives to *S. c* (%)**	**e-value**
HAT	Gcn5	**FGSG_00280 (FgGcn5)**	83	e-139
	Ada2	FGSG_12781	67	3e-87
	Ada3	**FGSG_00741 (FgAda3)**	45	2e-48
SPT	Ada1	FGSG_09909	40	1e-10
	Spt3	**FGSG_05091 (FgSpt3)**	63	9e-77
	Spt7	**FGSG_06291 (FgPCN)**	43	3e-91
	Spt8	**FGSG_02801 (FgSpt8)**	53	7e-57
	Spt20	FGSG_11969	35	8e-8
TAF	Tra1	FGSG_06089	56	0
	Taf5	FGSG_02010	45	5e-94
	Taf6	FGSG_09011	54	1e-77
	Taf9	FGSG_00864	56	1e-23
	Taf10	FGSG_01623	58	1e-24
	Taf12	FGSG_06044	36	1e-19
DUB	Ubp8	FGSG_07958	48	2e-55
	Sgf11	no hit	no hit	no hit
	Sus1	no hit	no hit	no hit
	Sgf73	**FGSG_05396 (FgSgf73)**	46	2e-11
	Sgf29	FGSG_04482	46	7e-15
	Chd1	FGSG_07102	55	0

Note: Putative orthologs of all SAGA complex subunits were identified in *F. graminearum*. Best hits in the *F. graminearum* genome database were identified using query protein sequences of the known *S. cerevisiae* SAGA complex members. Deletion mutants of SAGA complex in *F. graminearum* were highlighted in bold.

Supplementary Table 8. PCN susceptibility of deletion mutants of putative acetyltransferases in *F. graminearum*

Strain	Putative function	Growth inhibition rate at EC_{50} of PCN (%)
PH-1	-	(55.56±5.31)^{BC}
ΔFGSG_00280	**Histone acetyltransferase Gcn5**	**(90.54±2.50)**^{A}
ΔFGSG_02040	Histone acetyltransferase Elp3	(64.49±9.99)^{B}
ΔFGSG_06307	Histone acetyltransferase Spt10, putative	(56.00±7.61)^{BC}
ΔFGSG_04254	Histone acetyltransferase (Esa1), putative	(52.27±5.15)^{C}
ΔFGSG_08481	Histone acetyltransferase (Esa1), putative	(51.69±1.20)^{C}
ΔFGSG_06047	Histone acetyltransferase (MysT1), putative	(52.55±5.99)^{C}
ΔFGSG_08993	Histone Rtt109-type acetyltransferase	(55.00±3.51)^{BC}
ΔFGSG_07254	Histone acetyltransferase type b catalytic subunit	(56.04±5.99)^{BC}
ΔFGSG_07600	Histone acetyltransferase hpa2	(49.16±3.10)^{C}
ΔFGSG_10299	N-acetyltransferase complex Ard1 subunit, putative	(55.87±5.70)^{BC}
ΔFGSG_06376	N-acetyltransferase C complex catalytic subunit	(55.00±3.89)^{BC}
ΔFGSG_01906	N-acetyltransferase (Nat5), putative	(50.72±5.37)^{C}
ΔFGSG_05248	GNAT family acetyltransferase, putative	(57.30±6.12)^{BC}
ΔFGSG_05938	N-acetyltransferase activity	(57.87±4.92)^{BC}
ΔFGSG_06329	Acetyltransferase, GNAT family family	(57.78±3.76)^{BC}
ΔFGSG_12570	GNAT family acetyltransferase, putative	(55.00±3.51)^{BC}
ΔFGSG_00377	N-acetyltransferase, GNAT family, putative	(60.00±5.57)^{BC}
ΔFGSG_08357	GNAT family acetyltransferase, putative	(59.78±5.07)^{BC}
ΔFGSG_13873	GNAT family acetyltransferase, putative	(50.57±6.95)^{C}
ΔFGSG_04081	GNAT family acetyltransferase, putative	(52.51±3.10)^{C}
ΔFGSG_04543	Glucosamine 6-phosphate acetyltransferase	(58.99±4.29)^{BC}
ΔFGSG_03682	N-acetyltransferase activity	(54.14±5.38)^{BC}
ΔFGSG_07592	GNAT family acetyltransferase, putative	(53.37±4.61)^{BC}
ΔFGSG_09067	N-acetyltransferase	(51.93±3.87)^{C}
ΔFGSG_01985	Polyamine acetyltransferase	(53.13±2.22)^{BC}
ΔFGSG_09944	Acetyltransferase, GNAT family family	(53.83±3.08)^{BC}
ΔFGSG_06018	Acetyltransferase, GNAT family family	(53.04±1.96)^{BC}
ΔFGSG_02444	Acetyltransferase, GNAT family family	(53.33±4.75)^{BC}
ΔFGSG_11846	GNAT family acetyltransferase Nat4	(51.35±1.16)^{C}
ΔFGSG_00540	Acetyltransferase, GNAT family family	(50.00±3.85)^{C}
ΔFGSG_08082	N-acetyltransferase, GNAT family	(55.43±3.49)^{BC}

Note: Targets for gene deletion were chosen according to the Broad Institute *Fusarium* Comparative Database feature search for the genes harbored the

acetyltransferase domain. The deletion strains were obtained by homologous recombination using hygromycin as a selectable marker. All deletion strains were phenotypically tested the susceptibility to PCN at the EC50. The mycelium growth inhibition rate was presented as the mean ± s.d (n=3), and analyzed by Fisher's protected least significant difference (LSD) test at P=0.01. The most sensitive mutant, *ΔFgGCN5* (locus at *FGSG_00280*) was highlighted in bold.

Supplementary methods

Sequence Analysis of wheat microbiome

The Quantitative Insights Into Microbial Ecology (QIIME, v1.8.0) pipeline was employed to process the sequencing data (Sequence data has been deposited under Bioproject Accession PRJNA473402), as previously described [1]. Briefly, raw sequencing reads with exact matches to the barcodes were assigned to respective samples and identified as valid sequences. The low-quality sequences were filtered through following criteria [2]: sequences that had a length of <150 bp, sequences that had average Phred scores of <20, sequences that contained ambiguous bases and sequences that contained mononucleotide repeats of >8 bp. Paired-end reads were assembled using FLASH [3]. After chimera detection, the remaining high-quality sequences were clustered into operational taxonomic units (OTUs) at 97% sequence identity by UCLUST [4]. A representative sequence was selected from each OTU using default parameters. OTU taxonomic classification was conducted by BLAST searching the representative sequences set against the Greengenes Database[5] using the best hit [6]. An OTU table was further generated to record the abundance of each OTU in each sample and the taxonomy of these OTUs. OTUs containing less than 0.001% of total sequences across all samples were discarded. To minimize the difference of sequencing depth across samples, an averaged, rounded rarefied OTU table was generated by averaging 100 evenly re-sampled OTU subsets under the 90% of the minimum sequencing depth for further analysis.

Isolation culturable bacteria and identification ZJU60

At every stage of wheat, five samples were picked by a five-spot sampling method in a field, where 10 healthy and infected wheat heads with a 50% disease index were included in each sample. Briefly, each sample was homogenized with a sterilized mortar and pestle. Macerated samples were serially diluted in sterile 0.85% NaCl solution, and the resulting suspensions were plated onto LB agar plates supplemented with the fungicide carbendazim to prevent fungal growth. Plates were incubated at 25, 30 and 37 °C for 1-3 days.

For molecular identification of the BCA ZJU60, the cellular fatty acid analysis by gas chromatography was conducted as the manufacturer's

instructions [7]. Samples with a similarity index of 0.500 or higher with a separation of 0.100 between the first and second choice are considered good library comparisons.For identification on the MALDI (Matrix-Assisted Laser Desorption/ Ionization) Biotyper platform, ZJU60 was grown on the trypticase soy agar plate for 24 h at 30 °C, and the cells were then harvested and washed with sterilized water for protein extraction. Proteins were extracted from ZJU60 according to the ethanol/formic acid extraction method, and samples were then spotted in four technical replicates onto a MALDI target plate and analyzed by MALDI-TOF MS (Matrix-Assisted Laser Desorption/ Ionization Time of Flight Mass Spectrometry), according to the manufacturer's instructions [8]. Spectra were loaded into Biotyper software and identified against the MSP database library to obtain the Biotyper-derived scores. The genome of ZJU60 was sequenced at Beijing Novogene Bioinformatics Technology Co., Ltd. using the PacBio RSII sequencing platform (Pacific Biomarkers, Menlo Park, CA, USA). High-molecular-weight gDNA was extracted from overnight cultures using the SDS method. gDNA was fragmented and used for library construction with the PacBio SMRT Cell 10 kb Library preparation kit, and then sequenced utilizing PacBio RSII and C2 chemistry with 100X coverage according to the manufacturer's instructions. After filtering low quality reads using SMRT Analysis 2.3.0, the remaining reads were assembled to generate one contig without gaps, and then annotated using the NCBI Prokaryotic Genomes Automatic Annotation Pipeline.

Disease index and biocontrol efficacy calculation

Disease index in wheat heads was assessed with five evaluation classes, classified according to codes 0 to 4, which correspond to a percentage of wheat head surface showing FHB symptoms (0 = 0, 1 = 1 to 25, 2 = 26 to 50, 3 = 51 to 75, and 4 = >75%). Disease index (DI) of each plot was calculated using the formula [(Σ number of wheat heads in each class × each evaluation class)/(total number of wheat heads × 4)] ×100. Efficacy of each treatment was determined by applying Abbott's formula: [(DI of the negative control – DI of the treatment)/ DI of the negative control)] × 100%.

Chemo-genomic profiling assay

The yeast homozygous deletion pool (mixture of 4,653 different yeast gene deletion mutants) was available from Invitrogen (Cat. No. 95401.H1Pool, Carlsbad, USA). Yeast cells were cultured in YEPD (yeast extract/peptone/dextrose) under standard yeast growth conditions.

The screen of the homozygous pool was performed as described previously. Briefly, the frozen aliquot of the deletion pool was recovered for one generation and then added to fresh rich medium supplemented with 10 μg ml^{-1} PCN for ten more generations. Yeast DNA was extracted using a Yeastar Genomic DNA Kit (Zymo Research, Orange, USA). The DOWNTAGs were amplified using primers compatible with Illumina sequencing platform. Experiments for each compound were duplicated and the PCR products were quantified using Qubit 3.0 Fluorometer (Thermo Fisher Scientific, Waltham, USA). Equivalent parts of each product were mixed and sequenced on an Illumina HiSeq2500 system (Illumina, San Diego, USA).

Sequencing reads were mapped to yeast deletion collection barcodes provided by the manufacturer's online resource. We performed relaxed matching by allowing two mismatches. Reads with multiple hits were removed from further analysis. Similarly, the sample indices were matched with no more than one allowed mismatch. Counts for all samples were quantitatively normalized, and the chemical genetic interaction scores (CGIS) were calculated as a factor for drug sensitivity. The interaction networks for drug-sensitive (CGIS ≥ 3) and resistant (CGIS ≤-3) genes were extracted from the SGD database (http://www.yeastgenome.org/) and visualized by Cytoscape3.4.0 (http://www.cytoscape.org/). A total of 90 mutants with potentially altered sensitivity to PCN were recovered using this method. These 90 individual mutants and 345 their directly interacting gene mutants according to the SGD Database were streaked out from the yeast deletion strain collections (SRD, Scientific Research and Development GmbH) and their PCN sensitivities were tested on the YEPD medium supplemented with the PCN at 20 or 50 μg ml^{-1}.

Protein expression and purification

The ORFs of FgGCN5 and H3 were amplified using the cDNA of *F. graminearum* as template and subsequently cloned into the pET-22b(+) (6×His

tag) and pGEX-4T-3 vectors (GST tag), respectively. The recombinant plasmids were transferred into *Escherichia coli* BL21 for expression. For GST-H3 protein purification from *E. coli* BL21 harboring the expression vector, *E. coli* cells were incubated in LB medium containing 50 μg ml^{-1} ampicillin to an optional density at 600 nm of 0.4-0.6 at 37 °C in a shaker (200 rpm), and then the final concentration of isopropyl β-D-thiogalactoside (IPTG) ($5×10^{-4}$ M)was added. Four hours post induction at 16 °C, cells were collected by centrifugation at 9,600 rpm at 4 °C, washed in PBS (150 mM NaCl, 3 mM KCl, 10 mM Na_2HPO_4, 7 mM KH_2PO_4, pH 7.4), and disrupted using an ultrasonic processor (JY92-IIN, Scientz Biotech, China). After centrifugation for 30 min at 9,600 rpm at 4 °C, the supernatant containing whole-cell protein extract was collected and incubated with GST agarose (Prod#25236, Thermo Fisher Scientific, America) at 4 °C on a rotating wheel for 3 h, and the beads were washed twice with PBS and then eluted five times with elution buffer (10 mM glutathione dissolved in PBS). GST-H3 was dialyzed into 1×HAT buffer (50 mM Tris-HCl, pH 8.0, 50 mM KCl, 0.1 mM EDTA, 1mM DTT, 1 mM protease inhibitor, 5% glycerol) at 4 °C overnight. Whole-cell lysate of *E.coli* BL21 harboring the FgGcn5-6×His expression vector was also prepared according to the above procedures and then loaded onto a Ni-NTA column (His-TrapTM HP, GE Health Care, America)equipped with an AKTA purification system (GE Health Care, America). The columns were washed for 30 min with imidazole (dissolved in PBS) at concentrations ranging from 5 mM to 100 mM and eluted with 200 mM imidazole. The purified protein was desalted using a desalting column (HiPrepTM 26/10 Desalting, GE Health Care, America) installed in an AKTA purification system. Purified and desalted proteins were saved at -80 °C for future use.

Western blot assay

Fresh Fg mycelia (200 mg) were finely ground and suspended in 1 ml of extraction buffer (50 mM Tris-HCl, pH 7.5, 100 mM NaCl, 5 mM EDTA, 1% Triton X-100, 2 mM phenylmethylsulfonylfluoride (PMSF)) containing 10 μl of protease inhibitor cocktail (Sangon Co., Shanghai, China). After homogenization with a vortex shaker, the lysate was centrifuged at 10,000 *g* for 20 min at 4°C. Then, 100 μl of supernatant was mixed with an equal volume of 2 ×loading buffer and boiled for 5 min. The resulting proteins were

separated by 10% sodium dodecyl sulfate-polyacrylamide gel electrophoresis (SDS-PAGE) and transferred to Immobilon-P transfer membrane (Millipore, Billerica, MA, USA). To detect histone acetylation profiles of the wild-type strain with or without PCN treatment and the *ΔFgGCN5* mutant, all tested strains were grown in CM broth at 25°C with agitation (180 rpm) for 16 h. Wild-type mycelia were challenged with PCN at 15 or 25 μg ml^{-1} for 6 h before harvest. Proteins were separated by SDS-PAGE and transferred onto a polyvinylidene fluoride membrane with a Bio-Rad electroblotting apparatus. Then, the membranes were probed with anti-H2BK15ac (#61321,1:1000), -H2BK16ac (#39121, 1:10000), -H2BK46 ac (#39571, 1:10000), -H2BK120ac (#39119,1:2000), -H2BK12ac (#39669, 1:10000), -H3K4ac (#39382, 1:7500), -H3K9ac (#39137,1:10000), -H3K14ac (#39599, 1:10000), -H3K18ac (#39756, 1:7500), -H3K27ac (#39136, 1:10000), -H3K56 (#39281, 1:5000) , -H4K8 (#61103, 1:10000), -H4ac (pan-acetyl) (#39967,1:10000), -H3 (#61475,1:5000) or -H4 (#61299,1:2000) (Active Motif, La Hulpe, Belgium) or H2BK11(ab40975, 1:1000) (Abcam, Cambridge, USA) primary antibodies and a donkey anti-rabbit IgG-HRP secondary antibody (sc-2317, 1:10000) (Santa Cruz Biotech, Heidelberg, Germany). To evaluate Tri1-GFP expression under DON-inducing conditions, the tested strains were grown in TBI medium at 25°C with agitation (100 r.p.m) for 72 h. PCN was added at 24 h, and the fungicide carbendazim was used as a control to exclude the impact of growth arrest. The monoclonal anti-GFP ab32146 (Abcam, Cambridge, USA) antibody was used at a 1:5000 dilution for immunoblot analyses. Chemiluminescence was detected with an FDbio-Dura ECL Kit (FDbio Science, Hangzhou, China).The samples were also detected with the monoclonal anti-GAPDH antibody EM1101(Hangzhou HuaAn Biotechnology co., Ltd.) as a reference. The protein ladder (#26617, Thermo scientific) was loaded as the molecular weight marker. The experiment was conducted three times independently.

Molecular simulation analyses of PCN-FgGcn5 interactions

The binding positions of PCN in the active site of FgGcn5 were predicted using the MLSD program as described in previously reported studies [10, 11]. The structure of FgGcn5 was obtained using the Swiss model homology-modeling server [12] with the HAT domain sequence in GCN5 from *Saccharomyces cerevisiae* (PDB ID: 1YGH) as the template [13]. A grid box size of 80×80×80

dimensions with a spacing of 0.375 Å between the grid points was implemented and covered almost the entire GCN5-binding site. Lamarckian genetic algorithm and particle swarm optimization were used as searching method depending on the dimensionality of search space. Particle swarm optimization, a searching method inspired by bird flocking, was used for MLSD with relatively high dimensions of conformational space. The docking parameters were as follows: trials of 1000 dockings, the number of individuals in population was set to 300, maximum number of generations was 50,000 and other settings were set default.

Molecular dynamics (MD) simulations were used to verify the binding positions of PCN in the active site of FgGcn5. FgGcn5 molecules with one to three bound PCN molecules, determined by molecular docking, were used as initial structures. The charges of PCN were first obtained by the restrained electrostatic potential fitting technique based on the electrostatic potential computed by the Gaussian 09 at Hartree–Fock SCF/6-31G* level of theory. Subsequently, the topology and parameter files of PCN were generated using the antechamber module in the AMBER16 (Assisted Model Building with Energy Refinement 16) simulation package. Molecular mechanics parameters from the ff14SB [14] and GAFF2 force fields were assigned to the protein and ligands, respectively, using the LEaP module of the AMBER16 software package. These systems were all solvated in a box of TIP3P water molecules with a hydration shell of 10 Å. In addition, an appropriate number of counter ions was used to neutralize the systems.

MD simulations were carried out with the AMBER16 software package. Before MD productive simulation, we performed an equilibration protocol consisting of an initial minimization of the water molecules involving 5,000 steps: 2,500 steps for the steepest descent, and 2,500 steps in the conjugate gradient. The side chains of FgGcn5 were minimized by 5,000 steps for the steepest descent and 5,000 steps in the conjugate gradient. Subsequently, the water box including all atoms was minimized by 5,000 steps for the steepest descent and 5,000 steps. After minimization, the TIP3P water box was heated at a constant volume up to 300 K using a time constant for the heat bath with a coupling time of 100 ps. Equilibration was set at 300K and a constant pressure of 100 ps. Before MD production, 200 ps for the whole system were

equilibrated at a constant pressure of 1 bar. Finally, 100 ns of MD simulations were performed. During the simulations, non bonded interactions were cut off at 8.0 Å. Periodic boundary conditions were turned on at every step of the whole process. Particle mesh Ewald was also performed to address the long-range electrostatic interactions under periodic boundary conditions[15]. The SHAKE method was used to constrain hydrogen atoms and the time step was set to 2 fs. The coordinates were saved every 20 ps for the subsequent analysis.

Penetration ability of cellophane membranes assay

The penetration ability of each strain was examined on cellophane membranes, as described previously [16]. Briefly, each strain was grown on minimal medium (MM) covered with a cellophane membrane. The final concentration of PCN supplemented in MM plates was indicated. After 3 days of incubation at 25 °C, the cellophane membrane with the colony was removed from each plate. Penetrated mycelial growth on each plate was examined after incubation for another three days. The experiment was repeated three times.

Microscopy imaging

Green fluorescent signaling of Tri1-GFP in *F. graminearum* was visualized using a Zeiss LSM780 confocal microscope (Carl Zeiss AG, Germany). The mycelial morphologies of the wild-type strain and mutants in *F. graminearum* grown in CM or treated with PCN were visualized by SEM. Mycelia of each strain were harvested and fixed in 2% paraformaldehyde in 0.15 M phosphate buffer (pH 7.4) overnight. After fixation, mycelia were rinsed three times (15 min each) with 0.1 M PBS and refixed in 1% osmic acid for 2 h. Then, the samples were washed three times with PBS and dehydrated in graded ethanol solutions before critical point drying with CO_2. Bacterial biofilms, either ZJU60 alone or co-cultured with *F. graminearum*, and fungal penetration structures were also prepared as described above. To observe infection structures on wheat glumes, the spikelets were inoculated with fresh mycelia of wild-type, mutant or complemented strains and maintained in a high-humidity growth chamber for 48 h before fixation in 2% glutaraldehyde. Examination of samples was performed using a Hitachi S-4700 field emission scanning electron microscope (Hitachi, Tokyo, Japan).

Immunoblotting of Psl polysaccharide extracts

Bacterial cells were collected from 200 ml of WA and YEPD culture after 3 day of statical incubation at 30°C, respectively. Bacterial surface-associated Psl was extracted as previously described[17]. Briefly, the pellet was suspended in 0.9% NaCl and the cell surface-associated polymers were detached by mild sonication. The pellet was removed by centrifugation (25, 000 *g* for 1 h at 4 °C). From the supernatant, extracellular DNA was removed by precipitation with 25% ethanol and 0.1 M $CaCl_2$. The supernatant was dialysed, lyophilized, resuspended in water, and treated with proteinase K (final concentration 0.1 mg ml^{-1}) for 12 h at 37°C, followed by deproteinating with phenol (2×) and phenolchloroform. This crude polysaccharide extract was precipitated with ethanol, air-dry and dissolved in deionized water. Psl extracts were detected by immunoblotting with anti-Psl serum[18].

Supplementary References

1. Caporaso JG*, et al.* QIIME allows analysis of high-throughput community sequencing data. *Nat Methods* **7**, 335-336 (2010).
2. Gill SR*, et al.* Metagenomic analysis of the human distal gut microbiome. *Science* **312**, 1355-1359 (2006).
3. Magoc T, Salzberg SL. FLASH: fast length adjustment of short reads to improve genome assemblies. *Bioinformatics* **27**, 2957-2963 (2011).
4. Edgar RC. Search and clustering orders of magnitude faster than BLAST. *Bioinformatics* **26**, 2460-2461 (2010).
5. DeSantis TZ*, et al.* Greengenes, a chimera-checked 16S rRNA gene database and workbench compatible with ARB. *Appl Environ Microb* **72**, 5069-5072 (2006).
6. Altschul SF*, et al.* Gapped BLAST and PSI-BLAST: a new generation of protein database search programs. *Nucleic Acids Res* **25**, 3389-3402 (1997).
7. Sasser M. Identification of bacteria by gas chromatography of cellular fatty acids. MIDI Technical note #101: Newark, DE, USA, (1990).
8. Turvey ME, Weiland F, Meneses J, Sterenberg N, Hoffmann P. Identification of beer spoilage microorganisms using the MALDI Biotyper platform. *Appl Microbiol Biot* **100**, 2761-2773 (2016).
9. Yun Y, et al. Functional analysis of the *Fusarium graminearum* phosphatome. *New Phytol* **207**, 119-134 (2015).

10. Li H, Li C. Multiple ligand simultaneous docking: orchestrated dancing of ligands in binding sites of protein. *J Comput Chem* **31**, 2014-2022 (2010).
11. Wang Y*, et al.* Saturated palmitic acid induces myocardial inflammatory injuries through direct binding to TLR4 accessory protein MD2. *Nat Commun* **8**, 13997 (2017).
12. Biasini M*, et al.* SWISS-MODEL: modelling protein tertiary and quaternary structure using evolutionary information. *Nucleic Acids Res* **42**, W252-258 (2014).
13. Trievel RC*, et al.* Crystal structure and mechanism of histone acetylation of the yeast GCN5 transcriptional coactivator. *Proc Natl Acad Sci U S A* **96**, 8931-8936 (1999).
14. Maier JA, Martinez C, Kasavajhala K, Wickstrom L, Hauser KE, Simmerling C. ff14SB: Improving the Accuracy of Protein Side Chain and Backbone Parameters from ff99SB. *J Chem Theory Comput* **11**, 3696-3713 (2015).
15. Sagui C, Darden TA. Molecular dynamics simulations of biomolecules: long-range electrostatic effects. *Annu Rev Biophys Biomol Struct* **28**, 155-179 (1999).
16. Lopez-Berges MS, Rispail N, Prados-Rosales RC, Di Pietro A. A nitrogen response pathway regulates virulence functions in *Fusarium oxysporum* via the protein kinase TOR and the bZIP protein MeaB. *Plant Cell* **22**, 2459-2475 (2010).
17. Byrd MS*, et al.* Genetic and biochemical analyses of the *Pseudomonas aeruginosa* Psl exopolysaccharide reveal overlapping roles for polysaccharide synthesis enzymes in Psl and LPS production. *Mol Microbiol* **73**, 622-638 (2009).
18. Wang SW, Parsek MR, Wozniak DJ, Ma LYZ. A spider web strategy of type IV pili-mediated migration to build a fibre-like Psl polysaccharide matrix in *Pseudomonas aeruginosa* biofilms. *Environ Microbiol* **15**, 2238-2253 (2013).
