# Supplementary material for: Wheat microbiome bacteria can reduce virulence of a plant pathogenic fungus by altering histone acetylation
Source: Nat Commun. 2018 Aug 24;9:3429. doi: 10.1038/s41467-018-05683-7 (PMC6109063; doi:10.1038/s41467-018-05683-7)
Supplement: Supplementary file 3 — Description of Additional Supplementary Files [file 41467_2018_5683_MOESM3_ESM.pdf]

Description of Additional Supplementary Files

File Name: Supplementary Movie 1
Description: 100 ns MD simulation for one molecule of PCN with MD2.

File Name: Supplementary Movie 2
Description: 100 ns MD simulation for two molecules of PCN with MD2.

File Name: Supplementary Movie 3
Description: 100 ns MD simulation for three molecules of PCN with MD2.

File Name: Supplementary Data 1
Description: Relative fitness of each yeast mutant in the presence of PCN.

File Name: Supplementary Data 2
Description: Tested yeast mutants for PCN sensitivity.

File Name: Supplementary Data 3
Description: Gene IDs of partial tested mutants in *F. graminearum*.

File Name: Supplementary Data 4
Description: Penetration related genes in *F. graminearum*.

File Name: Supplementary Data 5
Description: Primers used in this study.
